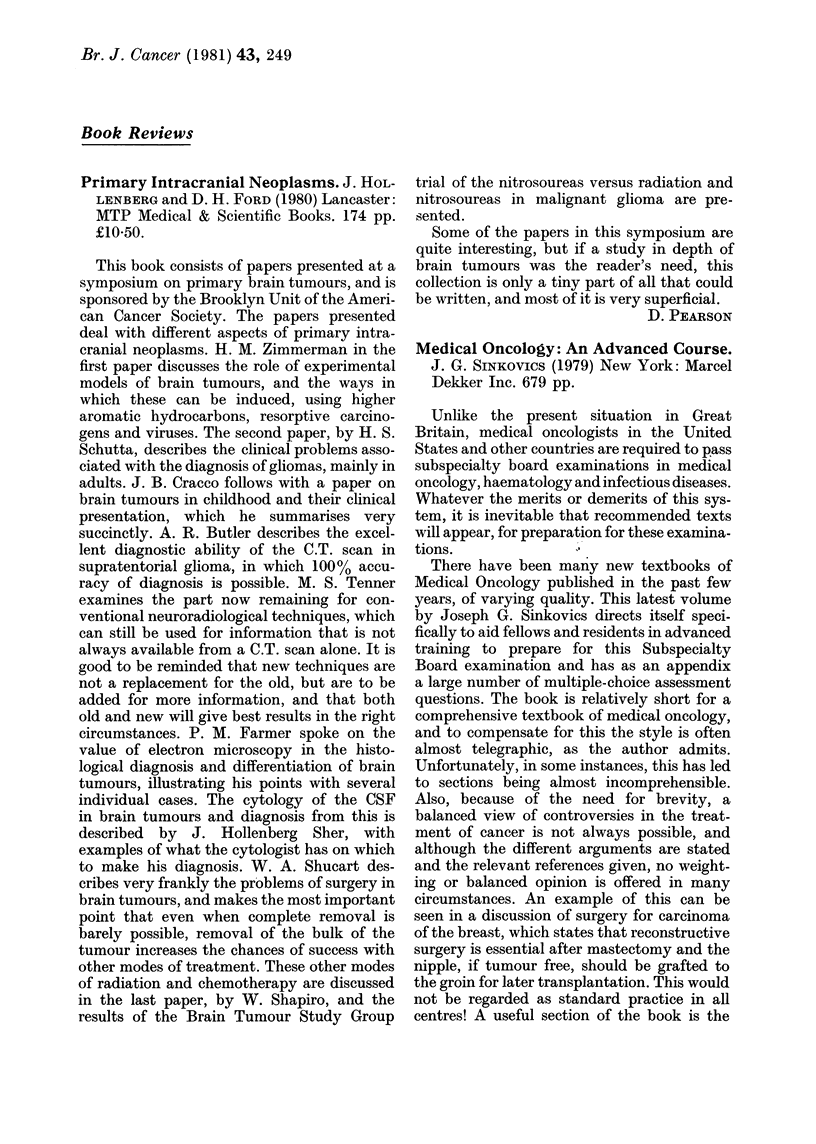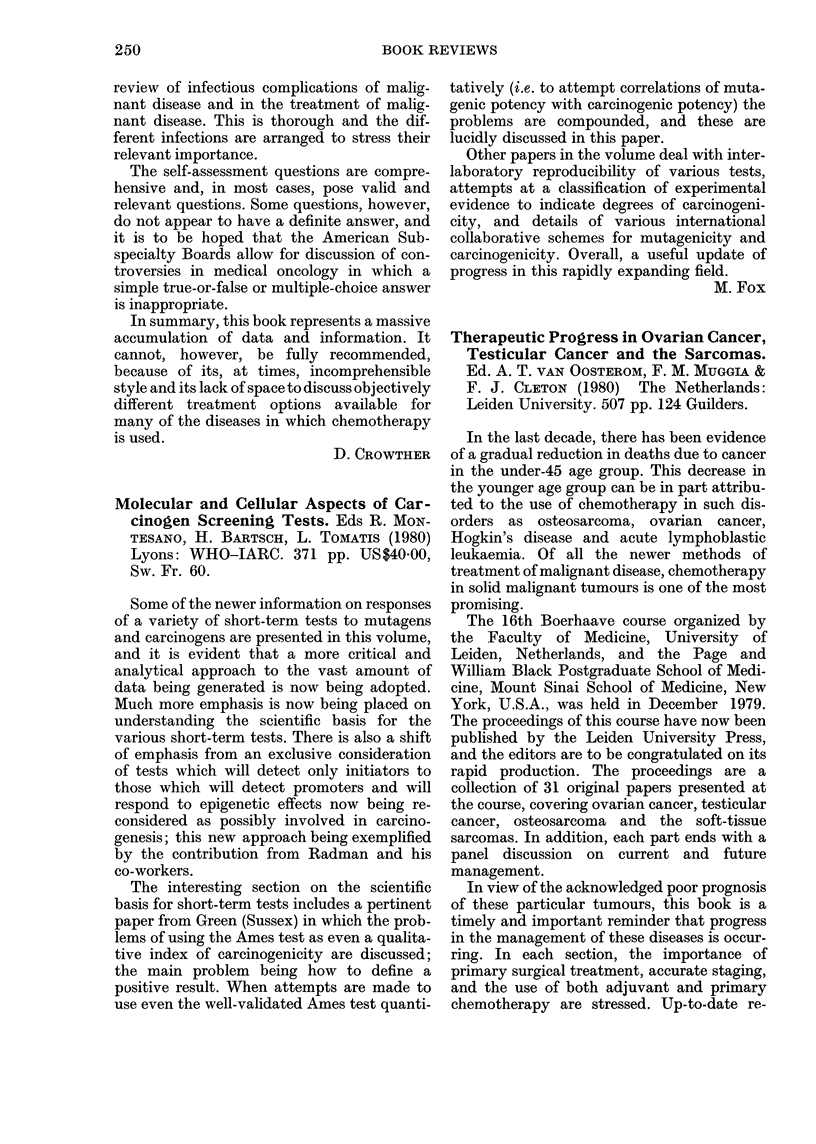# Medical Oncology: An Advanced Course

**Published:** 1981-02

**Authors:** D. Crowther


**Medical Oncology: An Advanced Course.** J. G. Sinkovics (1979) New York: Marcel Dekker Inc. 679 pp.

Unlike the present situation in Great Britain, medical oncologists in the United States and other countries are required to pass subspecialty board examinations in medical oncology, haematology and infectious diseases. Whatever the merits or demerits of this system, it is inevitable that recommended texts will appear, for preparation for these examinations.

There have been many new textbooks of Medical Oncology published in the past few years, of varying quality. This latest volume by Joseph G. Sinkovics directs itself specifically to aid fellows and residents in advanced training to prepare for this Subspecialty Board examination and has as an appendix a large number of multiple-choice assessment questions. The book is relatively short for a comprehensive textbook of medical oncology, and to compensate for this the style is often almost telegraphic, as the author admits. Unfortunately, in some instances, this has led to sections being almost incomprehensible. Also, because of the need for brevity, a balanced view of controversies in the treatment of cancer is not always possible, and although the different arguments are stated and the relevant references given, no weighting or balanced opinion is offered in many circumstances. An example of this can be seen in a discussion of surgery for carcinoma of the breast, which states that reconstructive surgery is essential after mastectomy and the nipple, if tumour free, should be grafted to the groin for later transplantation. This would not be regarded as standard practice in all centres! A useful section of the book is the review of infectious complications of malignant disease and in the treatment of malignant disease. This is thorough and the different infections are arranged to stress their relevant importance.

The self-assessment questions are comprehensive and, in most cases, pose valid and relevant questions. Some questions, however, do not appear to have a definite answer, and it is to be hoped that the American Subspecialty Boards allow for discussion of controversies in medical oncology in which a simple true-or-false or multiple-choice answer is inappropriate.

In summary, this book represents a massive accumulation of data and information. It cannot, however, be fully recommended, because of its, at times, incomprehensible style and its lack of space to discuss objectively different treatment options available for many of the diseases in which chemotherapy is used.

D. Crowther